# Probing the Influence of Surface Chemical Functionalization on Graphene Nanoplatelets-Epoxy Interfacial Shear Strength Using Molecular Dynamics

**DOI:** 10.3390/nano13020287

**Published:** 2023-01-10

**Authors:** Hashim Al Mahmud, Sagar U. Patil, Matthew S. Radue, Gregory M. Odegard

**Affiliations:** 1 Department of Mechanical Engineering, University of Kufa, Najaf 54001, Iraq; 2 Department of Mechanical Engineering-Engineering Mechanics, Michigan Technological University, 1400 Townsend Drive, Houghton, MI 49931-1295, USA

**Keywords:** ReaxFF, surface functionalization of graphene, wrinkled structure, rough topology, mechanical interlocking

## Abstract

In this work, a characterization study of the interfacial interaction between different types of graphene nanoplatelets and an epoxy matrix is computationally performed. To quantify the discrete mutual graphene–epoxy “interfacial interaction energy” (IIE) within the nanocomposite, molecular dynamics simulations with a reactive force field are performed on a localized model of the suggested nanocomposite. Pull-out molecular dynamics simulations are also performed to predict the interfacial shear strength between the two constituents. The results indicate a significant increase in interfacial adhesion of functionalized nanoplatelets with the hosting epoxy matrix relative to virgin graphene nanoplatelets. The obtained results also demonstrate a dramatic increase in the interfacial interaction energy (IIE) (up to 570.0%) of the functionalized graphene/epoxy nanocomposites relative to the unmodified graphene/epoxy nanocomposites. In the same context, the surface functionalization of graphene nanoplatelets with the polymer matrix leads to a significant increase in the interfacial shear strength (ISS) (up to 750 times). The reported findings in this paper are essential and critical to producing the next generation of lightweight and ultra-strong polymer-based nanocomposite structural materials.

## 1. Introduction

The development of aerospace structural materials has been going through a determined research effort for the last two decades [1,2,3]. Increasing the strength-to-mass density ratio has been one of the most crucial aims in the structural materials for the upcoming generation of aerospace vehicles. This goal can be most easily achieved with the use of advanced polymer matrix composite materials. More specifically, the remarkable engineering properties of graphene/epoxy nanocomposites have attracted the interest of both researchers and stakeholders. Therefore, such nanocomposites have been utilized to further improve the mechanical properties of traditional carbon fiber/epoxy composites by fabricating carbon fiber/graphene/epoxy hybrid composites [4,5,6,7,8,9,10]. Apart from structural materials, graphene-based materials find application in various fields such as ultra-narrow sensors for biomolecules detection [11] and electrochemical sensors for detecting harmful chemicals released into the air from flame retardants [12], modulators and detectors fabricated as a two-dimensional material [13], and water-treatment applications to clean-up impurities in water [14].

Graphene nanoplatelets (GNP) are widely utilized to reinforce polymer structures used in many engineering applications because of their remarkable mechanical, thermal, and electrical properties. The synthesizing process of GNP/epoxy nanocomposite materials requires professionality and a deep understanding of their molecular structure, especially when they are classified as advanced composites used in manufacturing aerospace and aeronautics structural components. Dealing with a wide range of key processing parameters in these nanocomposites, such as the type and curing degree of the epoxy resins, in addition to the size, concentration, and dispersion of the GNPs, is essential to optimize the mechanical properties and integrity of the bulk nanocomposite. The mutual interfacial interaction energy (IIE) is one of the most important factors used in determining the load transfer between nanocomposite constituents at the nanoscale. Fortunately, polymer matrices have shown a natural affinity to the reinforcements of carbon allotropes, including GNP. What is more, chemical modification (functionalization) of the GNP surface can further improve the mutual affinity with the hosting matrix [15,16,17].

The pull-out energy can be utilized to determine the shear strength or interfacial adhesion between two surfaces. Due to the difficulty and limitations of using standard experimental tools to fully access the pull-out energy, a reliable molecular dynamics (MD) computational tool called “pull-out MD” (POMD) simulations has shown its validity in addressing the interfacial shear strength (ISS) between nano-reinforcements (such as GNP and carbon nanotubes) and the hosting polymer matrix [18,19,20,21,22]. In this context, Lv et al. [18] performed POMD simulations using the “COMPASS force field” on graphene nanoplatelet/polymer nanocomposites to study the direct influence of the functionalization degree on the interfacial shear stress and bonding energy. They tested several configurations of functional groups to explore their influence on the interfacial binding of the modified graphene nanoplatelets with two polymer matrix systems. With respect to the polymer type, polyethylene (PE) provided improved mutual interaction with the modified nanoplatelets relative to that observed when using poly(methyl methacrylate) (PMMA). On the other hand, the functional group types produced different levels of interfacial bonding energy, which were arranged in the following order: -NH_2_ > -COOH > -OH > -F > -CH_3_. Finally, the study was concluded by specifying several factors which affect the mutual interaction energy between the constituents in the nanocomposite. These factors were identified by the used functional group types or their chemical structure, concentration, polarity, and their distribution on the nanoplatelet surface. However, the mutual shear stress level between the functionalized graphene nanoplatelets and polymer matrices was affected by another factor, which is referred to as the mechanical interlocking between them.

In another study, Lv et al. [19] investigated the characteristics of interfacial bonding between graphene and polyethylene (PE) using POMD simulations. The MD models were simulated using the COMPASS force field. Their study involved investigating the effect of three different chemical functional groups having molecular configurations of phenyl groups, -C_6_H_13_, and -C_2_H_4_(C_2_H_5_)_2_. The results indicated that the largest functional group type of -C_6_H_13_ produced the highest interfacial bonding energy between the constituents. This is because they were relatively well-embedded within the polymer matrix. However, the complex configuration of the -C_2_H_4_(C_2_H_5_)_2_ functional groups provided better mechanical interlocking, and, thus, the strongest shear force. This concludes that the size and molecular configuration of the functional group play an important role in controlling the interlocking mechanism and interfacial load transfer.

The POMD simulations were utilized by Jin et al. [23] to explore the interfacial mechanical properties between polyethylene (PE), as the polymer matrix, and nano-reinforcements of functionalized graphene sheets (FGS), where the letter “S” refers to five possible functional group types: -H, -O-, -OH, -NH_2_, -CH_3_. The reactive force field (ReaxFF), developed by Chenoweth et al. [24], was used to build the nanocomposite MD models and perform the required POMD simulations. The simulation results revealed that the ISS is highly governed by the molecular binding strength between the constituents. The researchers also noticed that some of the constraints in the functional groups represented by their configuration, size, and the coverage degree (number and distribution of the functional groups) were essential in determining the ISS. Furthermore, they were able to better access the influence of the polarity of the functional group on the interfacial interaction because of the use of the ReaxFF in their MD simulations. Accordingly, they determined that the polarity of the -O-, -OH, and -NH_2_ functional groups can produce higher electrostatic (Coulomb) energy relative to the -H and -CH_3_ functional groups, which are non-polar. Due to their high polarity, the epoxide -O- groups provided the highest level of interfacial binding energy. Thus, the epoxide -O- groups exhibited a traction effect on the PE chains that was comparable to the -CH_3_ functional groups, regardless of their poor interfacial binding.

The use of reactive force fields is critical for most MD simulations, and it could have benefits over using fixed-bond force fields, as it can efficiently capture the interactions of the intermolecular covalent bonding process occurring at the interface between the matrix molecules and the reactive functional groups in the nanofillers. In this context, Hou et al. [25] employed a reactive force field to study the interaction mechanisms in cement hydrates reinforced with graphene, as well as graphene oxide (GO). They specifically investigated the interfacial interaction associated with non-bridging oxygen atoms (NBO) in calcium silicate hydrate and the hydroxyl (-OH) groups in the GO. The major finding refers to an increase in the interfacial interaction and bridging of cracks when using GO nanoplatelets compared to as-received graphene. This is likely due to the relatively high polarity of GO, which creates interface counter-ions necessary to activate proton transfer with NBO and -OH and supports the formation of new H bonds. On the other hand, pristine graphene reinforcement produced a drop in the mechanical behavior because of the relatively weak bonding at the interface.

With the capabilities of POMD simulations to capture the characteristics of interfacial regions between nanofillers and polymer matrices, which are briefly presented above in addition to other relevant computational results in the literature [26,27,28,29,30,31,32,33,34], the current computational study has been established to probe and assess the IIE and ISS between epoxy and different types of GNPs. The effect of the surface functionalization of the graphene nanoplatelets on the IIE and the stick–slip behavior of the nanoplatelets relative to the epoxy matrix has been also investigated. The pull-out MD simulation data has been analyzed to specify the slipping onset region, which is necessary to capture the critical load transfer and, hence, estimate the ISS. Experimentally-driven MD models of functionalized graphene nanoplatelets have been used in this study to mimic real case studies rather than highly idealized MD models, which are commonly used in the literature. The modeling approach demonstrates the capabilities of ReaxFF in modeling different interfacial shearing scenarios including interfacial bond breaking. Finally, this computational study highlights the positive role of functionalization in improving the local ISS between graphene nanoplatelets and the surrounding epoxy matrix.

## 2. Materials and Modeling

This work utilizes MD samples for the interphase region of GNP/epoxy nanocomposites, which were previously modeled by Al Mahmud et al. [35]. Three independent nanocomposite samples were modeled using the MD simulator software, LAMMPS [36,37], with the parameter set of the ReaxFF force field, which was modified by Liu et al. [38]. These MD samples were previously established to investigate the effect of GNP functionalization on the mechanical properties of the nanocomposite. EPON828/DETDA reinforced with pristine GNP was simulated to represent the baseline case study of GNP/epoxy nanocomposite. The other two case studies of nanocomposites were simulated using the same epoxy matrix, yet this time reinforced with either highly concentrated graphene oxide (GO, where O represents two forms of oxygen-based functional groups, which are epoxide -O- and hydroxyl -OH) to form GO/epoxy nanocomposite, or with functionalized graphene oxide (FGO, where F represents two forms of nitrogen-based functional groups, which are Amine -NH_2_ and Amide -C(=O)-NH_2_) to represent the FGO/epoxy nanocomposite. The chemical compositions of these two case studies were modeled based on the data presented by Park et al. [39].

**Figure 1:**
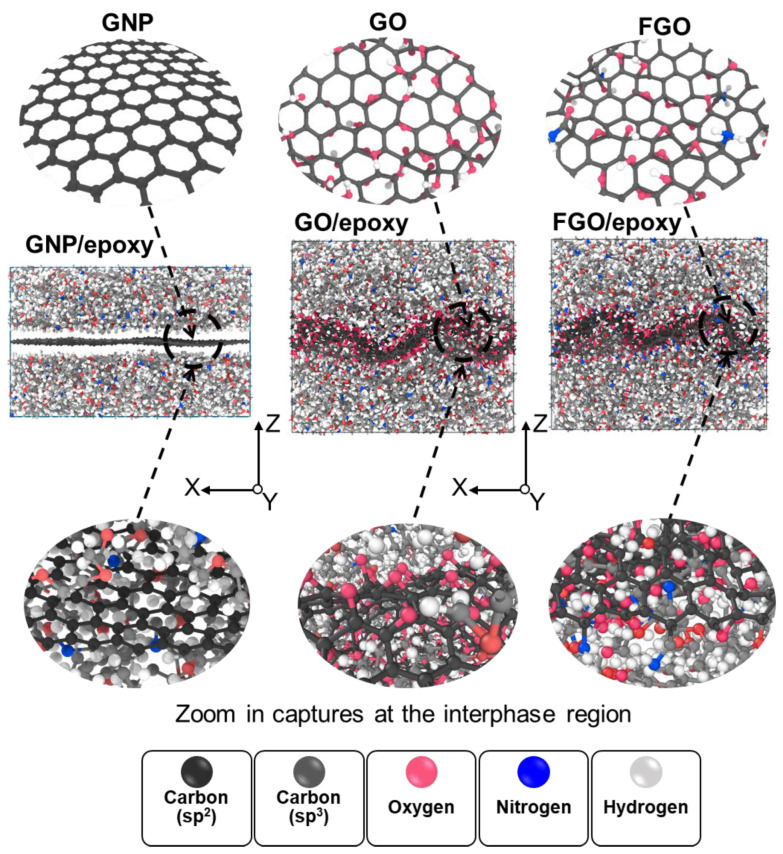
Local interphase region of GNP/, GO/, and FGO/epoxy nanocomposites, representative MD models.

**Figure 2:**
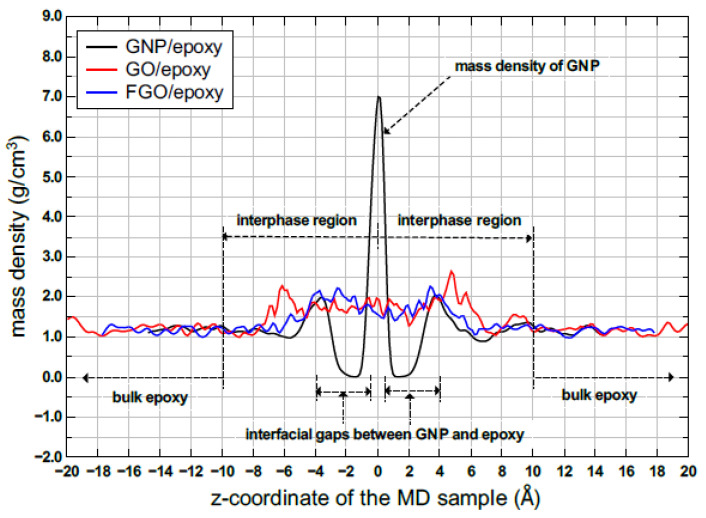
Representative spatial mass density distribution parallel to the z-coordinate for the MD models.

Five replicates of the MD models were established for every case study to consider the variation probability in the predicted mechanical properties. Representative models of the three nanoplatelet/epoxy (GNP/epoxy, GO/epoxy, and FGO/epoxy) systems are shown in Figure 1. The topology of the three graphene nanoplatelet types (GNP, GO, and FGO), along with zoomed-in captures at the interphase region between the nanoplatelets and epoxy, are also illustrated. Plots of the corresponding mass density distribution parallel to the z-coordinate of each MD model are shown in Figure 2. These plots are necessary to compare the effect of the functional groups on the molecular configuration of epoxy at the interface. An early observation can be inferred from Figure 1 and Figure 2, which refers to an agglomeration of epoxy molecules in the adjacent to GO and FGO nanoplatelets forming a clear and continuous region of relatively high-mass density across the interphase between the nanoplatelet and the matrix. For the GNP/epoxy, the agglomeration of epoxy molecules is separated from the GNP by a discontinuity region (gap). In both cases, the mass density of epoxy molecules decreases to the bulk level at around 10 Å from the nanoplatelets.

The atomistic data used in establishing the MD models are given in Table 1, which contains a detailed list of the number of atoms, number of functional groups, and parameters used in the simulated MD models. Table 2 includes the atomistic data and molecular structure parameters used in simulating the nanocomposite MD models. Additional details on the MD modeling and analysis can be found elsewhere [35,40].

## 3. Results and Discussions

Here is addressed two important correlative interfacial characteristics at the nanoplatelet/epoxy interphase region, which are investigated in two separate subsections. First, the prediction of the IIE between GNP and epoxy is investigated and discussed. The prediction of the ISS between GNP and epoxy is subsequently discussed.

### 3.1. Interfacial Interaction Energy (IIE)

The nanoplatelets/epoxy interfacial binding can be assessed using the IIE. The IIE for a nanoplatelet/epoxy nanocomposite can be estimated using Equation (1) using the potential energies of the nanoplatelets (PE_NP_), epoxy (PE_epo_), and the full nanocomposite MD model (PE_MD_) [7,41]:

IIE = PE_MD_ − (PE_NP_ + PE_epo_) (1)

**Figure 3:**
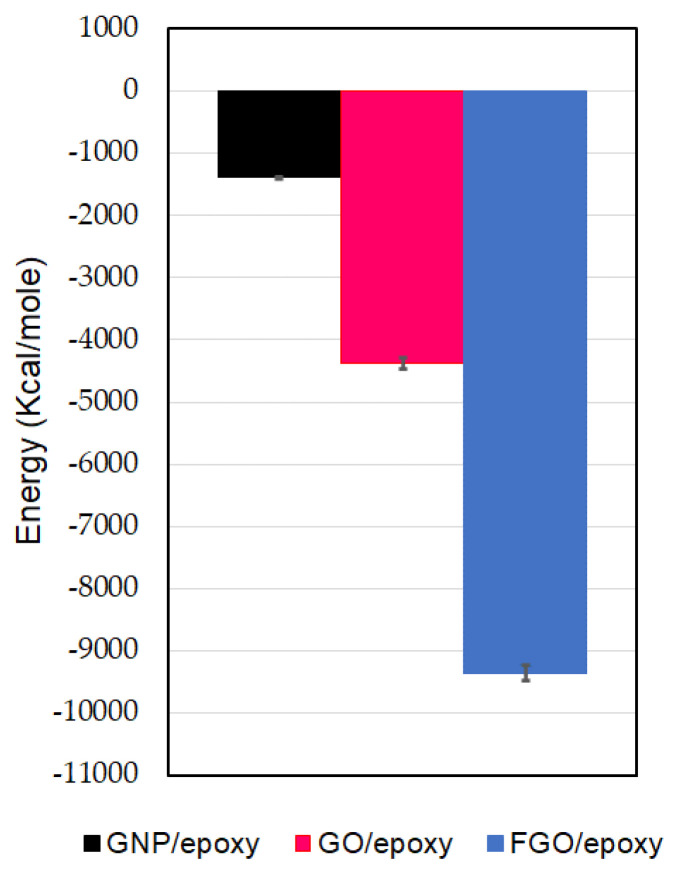
Interfacial interaction energy (IIE) of nanoplatelet/epoxy systems.

The levels of the estimated IIE for the GNP/epoxy, GO/epoxy, and FGO/epoxy systems are shown in Figure 3. For any of the three nanocomposite MD models, the reliable IIE level is obtained by taking the average of its predicted values from the MD replicates using the ReaxFF force field. Note that the stronger interaction between the nanoplatelet and hosting epoxy matrix is demonstrated by the larger negative value of IIE. Accordingly, the highest IIE among the three nanocomposites MD models is obtained for the FGO/epoxy MD model, which is estimated to be −9356 kcal/mol. The estimated IIE for the GO/epoxy model measures −4381 kcal/mole, which represents nearly half the interaction strength in the FGO/epoxy. Thereafter, the lowest interaction strength is obtained for the GNP/epoxy system, as its IIE measures −1389 kcal/mole. In other words, the IIE in the FGO/epoxy system exceeds that in the GO/epoxy and GNP/epoxy systems by 114% and 574%, respectively. Meanwhile, the IIE in the GO/epoxy system excels that in the GNP/epoxy system by 215%. These results indicate that introducing functional groups to the GNP surface results in a considerable increase in the IIE with the epoxy. Thus, the mutual nanoplatelet/epoxy adhesion can be considerably promoted with the functional groups, taking into consideration that the mutual interacting level is highly affected by the functional group type, its distribution, and functionalization degree.

**Figure 4:**
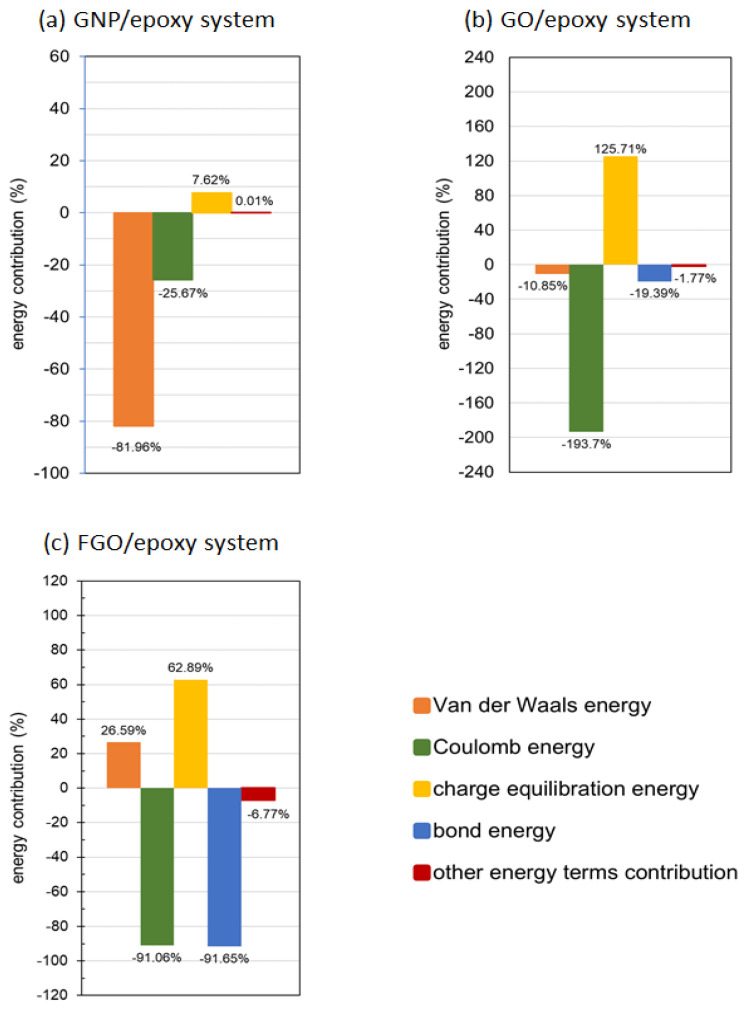
Energy term contributions in the predicted IIE for GNP/epoxy systems (**a**), GO/epoxy system (**b**), and FGO/epoxy system (**c**).

As the IIE involves different types of energy terms, which are established depending on the molecular system type and configuration, an analysis of the contribution percentages of the energy terms in the predicted IIE for each nanocomposite system is shown in Figure 4. Specifically, the column chart of energy term contributions shown in Figure 4a refers to the prime contribution percentage of van der Waals (vdW) energy term in the predicted IIE, which measures about −81.96% (attraction) in the GNP/epoxy system. The Coulomb energy contributed −25.67% (attraction) of the system energy, while the charge equilibration contributed +7.62% (repulsion). The other energy terms exhibited a neutral contribution that had no effect on the predicted IIE in the GNP/epoxy system.

Surface functionalization affects the molecular structure of the graphene lattice by changing its sp^3^ to sp^2^ ratio (Table 1), depending on the functionalization degree. This affects the contribution percentage of the energy terms and builds up different IIE levels relative to the GNP/epoxy system. In addition, the functional groups stimulate additional energy terms to have a role in building the mutual interaction, such as the bond energy term produced by the bridging bonds between the functionalized nanoplatelets and the epoxy matrix. For the GO/epoxy system, the column chart of energy term contributions shown in Figure 4b refers to the major contribution percentage of the Coulomb energy term in the predicted IIE, which measures −193.7% (attraction). An additional contribution percentage of −10.85% (attraction) is registered by the vdW energy term to establish the IIE in the GO/epoxy system, which is much lower than that observed in the GNP/epoxy system. The attraction effect in the IIE is further boosted by the −19.39% contribution percentage registered by a new energy term (bond energy term), which is promoted by the interfacial bridging hydrogen bonds (H-bond) between the GO and epoxy matrix. However, the attraction effect in the IIE is significantly reduced by the large repulsive contribution percentage of +125.71% from the charge equilibration. The other energies registered a small accumulative contribution of −1.74% (attraction) in the IIE.

For the FGO/epoxy system, the column chart of energy term contributions shown in Figure 4c indicates that the repulsive contribution percentage in the predicted IIE is registered by the vdW and charge equilibration energy terms of about +26.59% and +62.89%, respectively. An interesting observation refers to the transformation in the effect of the vdW energy term to show a repulsive contribution in contrast to the attractive contribution observed in the GNP/epoxy and GO/epoxy systems. However, the attraction effect in the IIE is mainly established by the Coulomb and bond energy terms, by about −91.00% each. An additional attractive contribution percentage of −6.77% is registered by the other energy terms in the FGO/epoxy system.

### 3.2. Interfacial Shear Strength (ISS)

**Figure 5:**
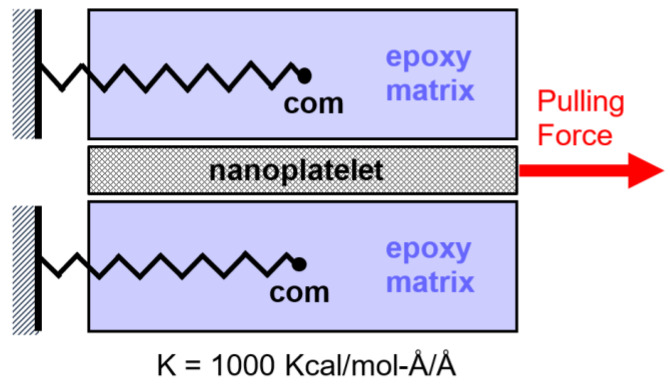
Mechanical setups for pull-out MD simulation.

POMD simulations [18,19,20,21,22,23] were utilized in this study to further investigate the effect of surface functionalization on the ISS between the nanoplatelets and epoxy matrix. The suggested hypothesis used to set up the mechanism of MD simulations is illustrated in Figure 5, which shows the loading conditions required to pull-out the nanoplatelets from the matrix. The simulation involves constraining the center of mass (com) of the matrix using virtual springs, whose other ends are attached to an imaginary fixed wall. This MD modeling step can be performed by using the “fix spring” LAMMPS command. The spring stiffness (K) was specified to be large enough (1000 kcal/mole-Å/Å) to hold the matrix while pulling out the nanoplatelets. The pulling action on the nanoplatelets is performed by using the “fix addforce” LAMMPS command. This command helps to simulate the application of a defined pulling force on each atom in the graphene lattice, which is selected here to be a function of the simulation time.

**Figure 6:**
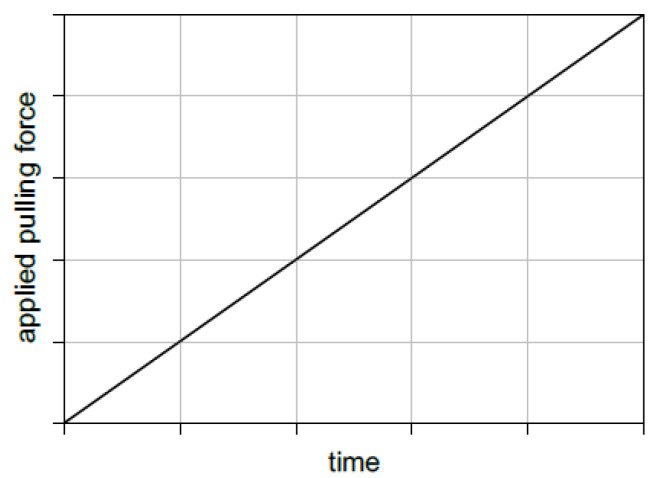
Applied pulling force profile as a function of simulation time.

The pulling process was proposed to be performed in a very steady slow manner. This is necessary to capture the critical value of the pulling force at which the relative interfacial slipping onset occurs between the nanoplatelet and the epoxy. Thus, the variable of the applied force was derived to be gradually increased with time in the MD simulation, which is obtained by using Equation (2):

F_pull_ = (F_max_ × t_step_)/t_total_ (2)

where F_pull_ represents the applied pulling force; F_max_ represents the maximum allowable pulling force; t_step_ represents the timestep used in the MD simulation; and t_total_ represents the total time of the MD simulation. This equation represents the linear (ramped-up) behavior of the applied pulling force whose profile is shown in Figure 6. Despite the simplicity of Equation (2) in which F_pull_ depends only on one variable (t_step_) and two constant values (F_max_ and t_total_), it is highly critical to specify the optimum values for these three terms. To clarify that, the increment in t_step_ has to be as small as possible to capture the threshold value of the pulling force in addition to being able to distinguish the stages in the obtained slipping profile. The total time required for the simulation process (t_total_) has to be as large as possible to satisfy the nanoplatelet slipping onset point and securely obtaining the entire slipping stage in the simulation process. To satisfy all of the above requirements, specifying the optimum value of F_max_ has to be somehow compatible with the other terms in Equation (2). An additional hidden term has to be considered in Equation (2) represented by the computational cost, which depends on the available computational resources.

**Figure 7:**
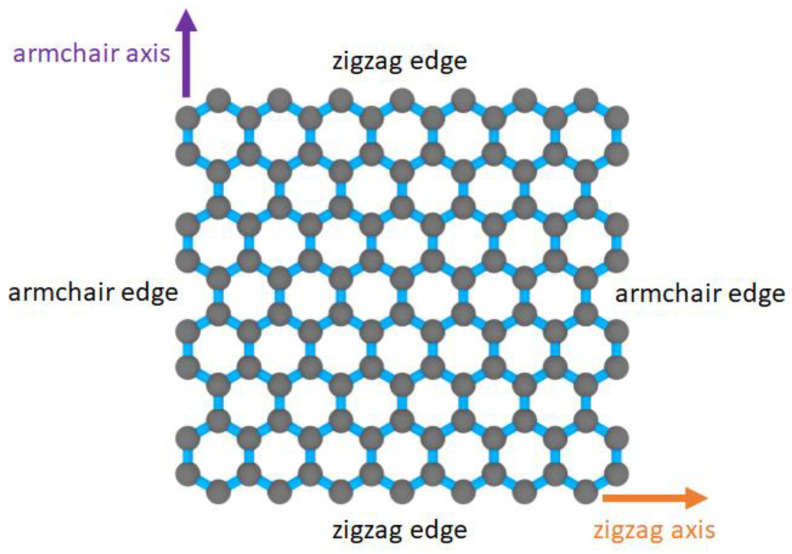
Hexagonal lattice structure in a graphene nanoplatelet.

Two important aspects were considered in the simulation process of all the MD models. First, it is necessary to activate the periodic boundary condition settings for the MD models to mimic a real-sized system. Second, both the zigzag and armchair directions in the graphene structure, which are illustrated in Figure 7, were considered as two unique directional axes in the MD-simulated pull-out. Accordingly, the pull-out simulations were performed along the two directions by placing the pulling force along each axis in separate MD simulations.

**Figure 8:**
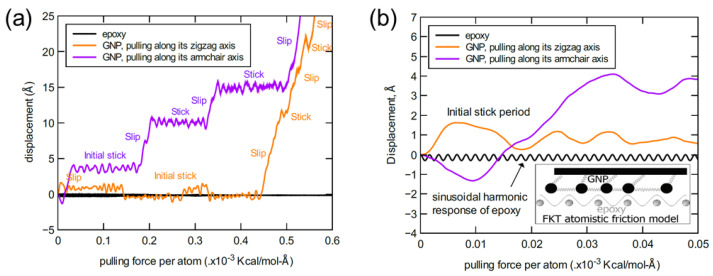
Representative response profile of GNP displacement relative to epoxy matrix as a function of the applied pulling force per lattice atom (**a**); detail of the early initial stick stage (**b**).

The following results were obtained after performing the pull-out simulations for the three nanoplatelet/epoxy systems. For a representative GNP/epoxy MD model, a displacement profile of the GNP as a function of the pulling force on at each carbon atom in the GNP lattice is shown in Figure 8. As mentioned previously, these displacement profiles were obtained considering that the pulling force is applied along the zigzag and armchair lattice directions of the GNP. The displacement profiles shown in Figure 8a demonstrate that the behavior of the initial slipping response along the zigzag axis is different relative to that observed while pulling along the armchair axis. By comparing the two responses, the initial sticking (slip resistance) stage along the zigzag axis demonstrates a larger slipping resistance (consumes larger potential energy) compared to that observed along the armchair axis. A zoomed-in capture at the early stage of the initial stick response in the slipping profile is shown in Figure 8b. The exaggerated response profile reveals the harmonic (sinusoidal wave-like profile) behavior of the epoxy matrix while resisting the GNP pull-out. Even with the heterogeneity in the molecular topology at the interface between the GNP and epoxy matrix, the interactive stick–slip behavior imitates the Frenkel–Kontorova–Tomlinson (FKT) model to represent the friction between two surfaces at atomistic scale [42].

Once the GNP starts its slipping after the initial sticking stage, pulling the GNP along the armchair direction demonstrates prominent stick-slip stages which form a stair-like profile. In contrast, a very short sticking stages can be observed once the GNP begins to slip along the zigzag axis. The relative difference in the stick–slip behavior is associated with the contrast in the slipping path along each of the two axes, which is governed by the energy surface topology of the aromatic lattice structure [43,44,45]. Thus, the energy required to jump from one energy well to another along the armchair direction is greater than that required along the zigzag direction. The stick–slip behavior in both cases keeps reproducing for a while as the pulling force increases with time. At a certain pulling force value, the pulling response reaches a new stage of a steady-state smooth slipping (no sticking) behavior. This stage was observed to occur at an average force threshold value of 0.478 ± 0.1 × 10^−3^ Kcal/mol-Å per carbon atom. On the other hand, the corresponding GNP displacement value was estimated to be around 25 Å, yet it was around 5 Å at the beginning of stick–slip behavior. Note that any of the two threshold values was obtained by estimating its average value over the MD replicate models and considering the two directional pulling axes in each replicate.

**Figure 9:**
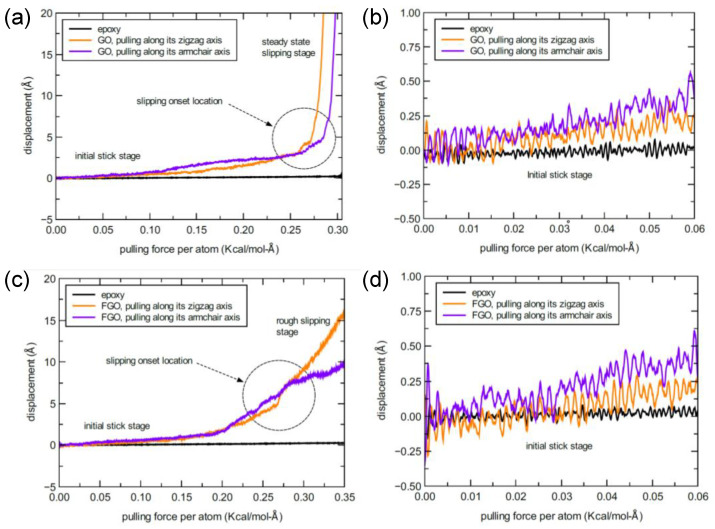
Representative response of GO (**a**) and FGO (**c**) displacement relative to epoxy as a function of the applied pulling force per carbon in the graphene lattice; detail of the early initial stick stage of GO (**b**) and FGO (**d**).

To assess the interfacial binding in the functionalized graphene nanoplatelet/epoxy systems, a quite similar MD simulation procedure was adopted. The results were also obtained and analyzed to be compared with the predictions above. As functionalized nanoplatelets exhibit a wrinkled structure and rough topology, in addition to the produced interfacial covalent and/or noncovalent bonding with the hosting epoxy matrix, both the GO and FGO were expected to yield larger pull-out resistance levels in comparison to that observed in the GNP/epoxy system. Figure 9 exhibits representative pull-out MD simulation responses in which the GO and FGO displacement relative to the epoxy were registered as a function of the applied force on each atom in the aromatic lattice and along the two directional axes.

For the GO/epoxy system, Figure 9a shows nearly identical responses, which were independently registered by pulling the GO nanoplatelet along its two directional axes. The response profile can be generally divided into an initial sticking stage and a slipping stage, which are separated by an inflection region. Given the response profiles of the MD replicates, a steady-state smooth slipping (no sticking) stage occurred at an average applied pulling force of 0.3 ± 0.022 Kcal/mol-Å per carbon in the nanoplatelet lattice. For the FGO/epoxy model, however, it is rather difficult to locate the slipping onset of the FGO from its response profile shown in Figure 9c. This is because the initial stick stage is followed by a persistent rough slipping stage, and there is no clear inflection point in the response between them. Zoomed-in captures at the early initial stick stage of GO and FGO response profiles are provided in Figure 9b and Figure 9d, respectively. The exaggerated profiles shown in these figures reflect the strong interfacial interaction, which is clearly different than that observed in GNP/epoxy system shown above in Figure 8b, which reflects the weaker non-covalent interfacial interaction. The rough slipping feature that dominates the response profile of the FGO is recognized by the large amount of fluctuation, which extends beyond the slipping onset region. This fluctuated response is most likely originated due to the mechanical interlocking between the wrinkled FGO and the epoxy matrix, in addition to interfacial bond scission. Given all the displacement response profiles of the FGO along the two axes and for the MD replicates, the slipping onset point occurred at an average threshold applied pulling force of 0.361 ± 0.05 Kcal/mol-Å per carbon in the nanoplatelet lattice, which is slightly larger than that for the GO. For both nanoplatelet types, however, the corresponding displacement threshold value was estimated to occur at a distance of around 5 Å. Beyond this displacement value, the slipping process of the nanoplatelets becomes slightly easy/smooth and irreversible, while the pulling force is gradually increased.

**Figure 10:**
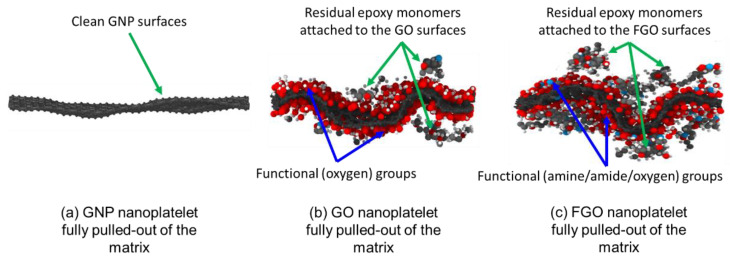
Representative MD screenshots of the fully pulled-out (detached) GNP (**a**), GO (**b**), and FGO (**c**) nanoplatelets, which were taken at specific time steps of the POMD simulations.

From the visual perspective, Figure 10 exhibits representative MD screenshots of the three nanoplatelets that were taken at specific, critical time steps of the POMD simulations. The screenshots were deliberately taken to investigate the nanoplatelet surface failure, which was when the nanoplatelets were completely detached and fully pulled-out of the matrix. For the GO nanoplatelet shown in Figure 10b, a few epoxy monomers/monomer parts/atoms still adhered to the nanoplatelet surfaces after detaching. However, the fracture surface mostly passed along the interface region between the functional (oxygen) groups and the epoxy matrix.

For the FGO nanoplatelet shown in Figure 10c, more epoxy monomers/monomer parts/atoms were still covalently attached to the FGO nanoplatelet surface. This indicates that the crack growth, to some extent, passed through the matrix monomers in the interphase region (refer to Figure 1 for definition of interphase). This can be attributed to the strong covalent bonding between the functional groups and the epoxy matrix, thus forcing failure to occur in some parts of the interphase region. Therefore, the fracture surface involved a mixture of epoxy monomers and functional groups. In contrast, this failure pattern did not apply to the GNP/epoxy nanocomposite, as the detaching (fracture surface) of the polymer molecules occurred entirely at the interfacial gap between the GNP and the epoxy matrix. Intuitively, the noncovalent interfacial binding between the GNP and epoxy represents the weakest area in the nanocomposite. Therefore, the fracture surface is most likely to be clean in this case, that is, no matrix material remains attached to the GNP surfaces, as shown in Figure 10a.

**Figure 11:**
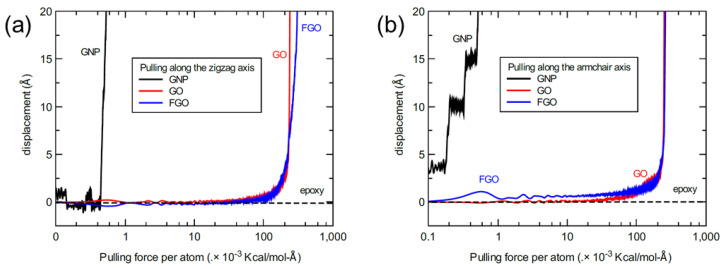
Representative compiled profiles of the nanoplatelets’ displacement relative to the epoxy, plotted as a function of the corresponding applied pulling force per nanoplatelet carbon; (**a**) pull-out of the nanoplatelets parallel to the zigzag axis and (**b**) armchair axis.

To accomplish analyzing the difference in the pulling response between the three nanoplatelet types, Figure 11 shows representative profiles of the slipping responses compiled together in one graph. In order to accommodate large-scale data points in one graph and maintain the behavioral profile for each response, the predicted displacement values for each nanoplatelet were plotted versus the logarithmic values (log_10_) of the applied pulling force per carbon in the nanoplatelet lattice and parallel to the two directional axes. Once again, the particular different behavior in the GNP slipping profile is recognized at the initial stick stage, which is highly sensitive to the pulling direction. In contrast, all the slipping profiles of GO and FGO are closely analogous by using the logarithmic scale and demonstrate less sensitivity to the pulling direction. More importantly, both the GO and FGO require larger magnitudes of the applied pulling force to fulfill their slipping onset, which is relatively much lower (in the range of two to three orders of magnitude) for the GNP. The nearly identical mechanical behavior observed in the slipping of the GO and FGO ensures that the nanoplatelet-matrix ISS is likely to be heavily influenced by the interlocking between the two constituents, which is established by the wrinkled and rough surfaces of the functionalized nanoplatelets.

Given that ISS is the pulling force threshold registered at the onset of slipping, Table 3 provides a list of all the predicted pulling force threshold values per nanoplatelet carbon atom. These threshold values were estimated for the five MD replicates along the two directional axes, and for the three nanocomposite systems. An averaged force threshold value was obtained parallel to each directional axis and then averaged over the two directional axes for each nanocomposite system. The possible uncertainty values corresponding to the standard deviation in the force threshold values were also determined.

In addition to its importance in estimating the ISS, the pulling force threshold value is essential to assess the static interfacial friction between the nanoplatelet and the hosting epoxy matrix. The ISS can be basically determined by multiplying the overall applied pulling force threshold value averaged over the MD replicates of the nanocomposite system by the number of carbon atoms in the nanoplatelet. Thus, as the graphene nanoplatelet lattice was modeled with 836 carbon atoms, the ISS in the GNP/epoxy system is estimated to be about 0.4 kcal/mole-Å. This is significantly less than the ISS in the GO/epoxy system, where its average value was estimated to be about 250 kcal/mole-Å. The FGO/epoxy system, however, surpasses the other nanocomposite systems by registering the highest ISS with an average value estimated to be about 300 kcal/mole-Å. In other words, the surface functionalization of the GNP resulted in a significant enhancement in the produced ISS in the GO/epoxy and FGO/epoxy systems over the GNP/epoxy system by about 625 and 750 times, respectively. The surface functionalization of the GNP resulted in a tremendous enhancement in the produced ISS in the GO/epoxy and FGO/epoxy systems over the GNP/epoxy system, by about 625 and 750 times, respectively. Due to the covalent interfacial bonding involving the highly reactive amine/amide functional groups in the FGO and the hosting epoxy, the ISS of the FGO/epoxy system is larger than that of the GO/epoxy system by about 20%.

Based on the ISS values, which are estimated for the three nanocomposite systems, it is clear that the functionalization of graphene nanoplatelets can substantially enhance adhesion at the nanoplatelet–matrix interface. Thus, the bulk material strength and toughness are implicitly promoted. The predicted results confirm that the degree of improvement in the material ISS is highly reliant on the composition and quantity of the functional groups. This can be logically recognized by the additional improvement in the ISS of the FGO/epoxy system over the GO/epoxy system, which is attributed to the interfacial covalent bonds established between the amine/amide functional groups and the epoxy matrix. In general, producing a nanocomposite with a robust molecular structure is governed by establishing an interface with covalent and noncovalent bonding. The interface interlocking is stimulated by the wrinkled and rough topology of the GO and FGO, and results in improvement of the material robustness. These factors qualify the localized interphase regions of the functionalized nanoplatelet/epoxy to serve as strong toughening zones within the bulk composite material.

## 4. Conclusions

This computational study utilizes experimentally informed nanocomposite MD models to address the effect of surface functionalization on the graphene/epoxy interfacial binding using POMD simulations. It has been established that introducing functional groups to the graphene surface can significantly improve both IIE and the ISS for the simulated nanocomposites. The outcomes from the simulated nanocomposite MD models refer to the merit of using GO rather than GNP reinforcements which is demonstrated by increasing the IIE up to 215% and increasing the IIE up to 625 times in the nanocomposite. This significant promotion in the material interfacial properties was primarily originated due to the high concentration of oxygen groups bonded to the graphene nanoplatelet. Substituting some of the oxygen functional groups with highly reactive amine and amide functional groups resulted in further increasing the IIE up to 570% and the ISS up to 750 times in FGO/epoxy relative to GNP/epoxy system. Such reactive functional groups have been found to promote the nanoplatelet-matrix interfacial adhesion by establishing supplementary covalent and noncovalent interaction energies. Other additional factors represented by the nanoplatelets wrinkling and rough topology of GO and FGO have been found to enhance the interference between the functionalized nanoplatelets and the hosting matrix. This, in turn, promotes the interlocking mechanism between the two constituents at the interphase region. Consequently, locally promoting the nanoplatelet-matrix interfacial binding is implicitly essential in establishing the bulk-level strength and toughness of the nanocomposite material.

## Figures and Tables

**Table 1:** Modeling of the GNP, GO, and FGO nanoplatelets.

Graphene Nanoplatelet Type	Carbon Atoms in the Graphene Lattice	Oxygen Functional Groups Attached to Surface of the Graphene Lattice		Nitrogen Functional Groups Attached to the Surface of the Graphene Lattice			Total Number of Atoms	sp^3^/sp^2^ Ratio	C:O Ratio
		-O-	-OH	-NH_2_	-C(=O)-NH_2_	-N-			
**GNP**	836	-	-	-	-	-	836	-	-
**GO**	836	271 × 1	271 × 2	-	-	-	1649	0.97	1.5
**FGO**	825	207 × 1	207 × 2	34 × 3	12 × 5	11 × 1	1619	0.80	1.9

**Table 2:** Atomistic data and modeling parameters used in simulating the nanocomposite MD models.

Parameter/Nanocomposite	GNP/Epoxy System	GO/Epoxy System	FGO/Epoxy System
total number of all atoms	7028	7841	7811
degree of crosslinking between monomers	~80%	~80%	~80% ^1^
molecular mass density (ρ), g/cm^3^	1.27 ± 0.01	1.42 ± 0.01	1.38 ± 0.01
nanoplatelet content (wt%)	19.6 ± 0.00	31.5 ± 0.00	29.5 ± 0.00
nanoplatelet content (vol%)	11.4 ± 0.09	19.2 ± 0.24	18.2 ± 0.17
nanoplatelet waviness factor (wf)	1.0	0.9	0.9

^1^ 60% of the overall degree of cross-linking is within the epoxy monomers network, and the remaining 20% is between epoxy monomers and FGO.

**Table 3:** Threshold values of pulling force in Kcal/mol-Å, predicted per carbon atom in the nanoplatelet lattice.

MD Models:	GNP/Epoxy		GO/Epoxy		FGO/Epoxy	
Directional Axis:	Zigzag	Armchair	Zigzag	Armchair	Zigzag	Armchair
model #1	0.679 × 10^−3^	0.642 × 10^−3^	0.276	0.310	0.358	0.400
model #2	0.445 × 10^−3^	0.398 × 10^−3^	0.282	0.295	0.309	0.344
model #3	0.445 × 10^−3^	0.413 × 10^−3^	0.319	0.269	0.359	0.378
model #4	0.441 × 10^−3^	0.410 × 10^−3^	0.288	0.313	0.300	0.311
model #5	0.437 × 10^−3^	0.472 × 10^−3^	0.339	0.310	0.384	0.464
average	(0.489 ± 0.106) × 10^−3^	(0.467 ± 0.102) × 10^−3^	0.301 ± 0.027	0.300 ± 0.018	0.342 ± 0.036	0.379 ± 0.058
average	(0.478 ± 0.100) ×10^−3^		0.300 ± 0.022		0.361 ± 0.050	

Note: These values were evaluated at the onset of steady-state smooth (no-stick) slipping stage of the nanoplatelets.

## Data Availability

The data are currently being used for further studies; thus, they are not publicly available. Please contact the corresponding author to request the data presented in this study.
